# Integrative nomogram of intratumoral, peritumoral, and lymph node radiomic features for prediction of lymph node metastasis in cT1N0M0 lung adenocarcinomas

**DOI:** 10.1038/s41598-021-90367-4

**Published:** 2021-05-24

**Authors:** Sushant Kumar Das, Ke-Wei Fang, Long Xu, Bing Li, Xin Zhang, Han-Feng Yang

**Affiliations:** 1 Department of Interventional Radiology, Affiliated Hospital of North Sichuan Medical College, 63 Wenhua Road, Nanchong, 637000 Sichuan People’s Republic of China; 2 Department of Interventional Radiology, West China School of Public Health and West China Fourth Hospital, Sichuan University, 18 South Renmin Road, Chengdu, 610000 Sichuan People’s Republic of China; 3 GE Healthcare, Life Sciences, No. 1 Tongji South Road, Beijing, 100176 People’s Republic of China

**Keywords:** Cancer, Cancer imaging

## Abstract

Radiomics studies to predict lymph node (LN) metastasis has only focused on either primary tumor or LN alone. However, combining radiomics features from multiple sources may reflect multiple characteristic of the lesion thereby increasing the discriminative performance of the radiomic model. Therefore, the present study intends to evaluate the efficiency of integrative nomogram, created by combining clinical parameters and radiomics features extracted from gross tumor volume (GTV), peritumoral volume (PTV) and LN, for the preoperative prediction of LN metastasis in clinical cT1N0M0 adenocarcinoma. A primary cohort of 163 patients (training cohort, 113; and internal validation cohort, 50) and an external validation cohort of 53 patients with clinical stage cT1N0M0 were retrospectively included. Features were extracted from three regions of interests (ROIs): GTV; PTV (5.0 mm around the tumor) and LN on pre-operative contrast enhanced computed tomography (CT). LASSO logistic regression method was used to build radiomic signatures. Multivariable regression analysis was used to build a nomogram. The performance of the nomogram was assessed with respect to its calibration, discrimination, and clinical usefulness. The discriminative performance of nomogram was validated both internally and externally. The radiomic signatures using the features of GTV, PTV and LN showed a good ability in predicting LN metastasis with an area under the curve (AUC) of 0.74 (95% CI 0.60–0.88), 0.72 (95% CI 0.57–0.87) and 0.64 (95% CI 0.48–0.80) respectively in external validation cohort. The integration of different signature together further increases the discriminatory ability: GTV + PTV (GPTV): AUC 0.75 (95% CI 0.61–0.89) and GPTV + LN: AUC 0.76 (95% CI 0.61–0.91) in external validation cohort. An integrative nomogram of clinical parameters and radiomic features demonstrated further increase in discriminatory ability with AUC of 0.79 (95% CI 0.66–0.93) in external validation cohort. The nomogram showed good calibration. Decision curve analysis demonstrated that the radiomic nomogram was clinically useful. The integration of information from clinical parameters along with CT radiomics information from GTV, PTV and LN was feasible and increases the predictive performance of the nomogram in predicting LN status in cT1N0M0 adenocarcinoma patients suggesting merit of information integration from multiple sources in building prediction model.

## Introduction

Non-small-cell lung cancer (NSCLC) typically metastasizes to hilar and mediastinal lymph nodes (LNs)^1^. While lobectomy with systematic lymph node dissection (LND) is a standard procedure for NSCLC^2,3^, lobe-specific selective lymph nodal dissection (SLND) has been used as an alternative to LND in patients with early stage NSCLC in order to reduce perioperative complications; especially for elderly patients or the patients with impaired pulmonary functions^4,5^. However, as most of the early NSCLC patients did not have metastasis to all of the systematic LNs, systematic LND leads to invalid LND^5,6^. On the other hand, as patients clinically diagnosed with cN0 LNs pre-operatively might have lymph node metastases pathologically. SLND overlook these LNs which would later progress and result in a poor prognosis^7–9^. Therefore, it is important to predict lymph node metastasis as accurately as possible, in order to help formulate individualized treatment strategies.

Currently, high-resolution computed tomography (HRCT), integrated Fluorine-18 2-fluoro-2-deoxy-d-glucose positron emission tomography/computed tomography (FDG-PET/CT), and mediastinoscopy or endobronchial ultrasound-guided transbronchial needleaspirate (EBUS-TBNA) are mainly used to predict lymph node metastasis in NSCLC in clinical practice^10^. However, mediastinoscopy or EBUS-TBNA are invasive methods which are costly and possess several serious complications^11,12^. FDG-PET/CT is not a commonly done for early-stage tumors with no enlarged LNs on CT imaging in China due to its economic burden for patients^13^. Therefore, CT is still the mainstay for screening lymph node status in early stage NSCLC, but it is limited by low sensitivity in the evaluation of small metastatic LNs. Thus, improvement in diagnostic performance of CT imaging in discriminating LN status in patients with early-stage NSCLC is of paramount importance.

Radiomics has emerged as a non-invasive method to derive quantitative features from medical imaging which has displayed great promise in oncological practice^14–19^. Studies have reported higher sensitivity and specificity for CT radiomics in predicting lymph node metastasis in patients with NSCLC^13,20–23^. Most of these studies principally focused on the evaluation of either the primary pulmonary nodule^13,20,21^, or the LN alone^22,23^. Theoretically, combined CT texture information from tumor as well as LN would be more reliable in assessing lymph node status. Furthermore, more recently peritumoral microenvironments has been found to offer its utility for clinical evaluation of tumor aggressive biological behavior^24–26^. Moreover, studies analyzing LN feature have smaller sample^22,23^ and analyzed larger LN ignoring normal sized LN^22,23^. He et al.^20^ did not perform whole tumor analysis instead extracted features from only large cross-sectional area of the tumor. Most studies are also limited by lack of standardization of imaging which might have led to batch effect^13,20–23^. In addition, aforementioned studies did not perform external validation, instead different types of internal validation (samples from single institute) such as cross-validation^13,21,22^ or temporal validation^20^ method was used. Herein the present study, we aim to determine the integrative value of clinical features and CT radiomic data extracted from gross tumor volume (GTV), peritumoral volume (PTV), and LNs, in prediction of LN metastasis in cT1N0M0 lung adenocarcinoma patients.

## Materials and methods

### Ethics approval and consent to participate

The current study was conducted in accordance with the 1964 Declaration of Helsinki^27^ and was approved by the Institutional Review Board of Affiliated Hospital of North Sichuan Medical College (Nanchong, China). Written informed consent was obtained from all the patients.

### Patients

Patients with clinical stage cT1N0M0 lung adenocarcinoma, according to the 8th Edition of Tumor-Node-Metastasis (TNM) classification^28^, who underwent surgical resection and systematic LN dissection at the Interventional Radiology Department of the Affiliated Hospital of North Sichuan Medical College (Nanchong, China) between January 2016 and March 2019, were included in the present retrospective study as a primary cohort. The criteria for enrollment were as follows: solitary pulmonary nodule in clinical stage T1 based on CT imaging; no enlarged lymph nodes (i.e., short diameter of LN < 10.0 mm on CT imaging); had undergone lobectomy or sub-lobectomy with systematic lymph node dissection; patients ≥ 18 years of age; and had an Eastern Cooperative Oncology Group performance status of 0 or 1^29^. The exclusion criteria were: patients with history of extra pulmonary malignancy; neoadjuvant chemotherapy or radiotherapy before surgery; and lack of CT imaging or CT artifacts. Finally, a primary cohort after applying the inclusion and exclusion criteria consisted of 163 patients with 163 tumors. Patients in the primary cohort were stratified based on the lymph node status and then randomly divided in a ratio of 7:3 into training and internal validation cohort. Training cohort consisted of 113 patients [mean age ± standard deviation (SD) 60.41 ± 9.90; range 41–83; male 66; female 47] while internal validation cohort consisted of 50 patients (mean age ± SD 62.87 ± 10.53; range 39–81; male 32; female 18).

**Figure 1:**
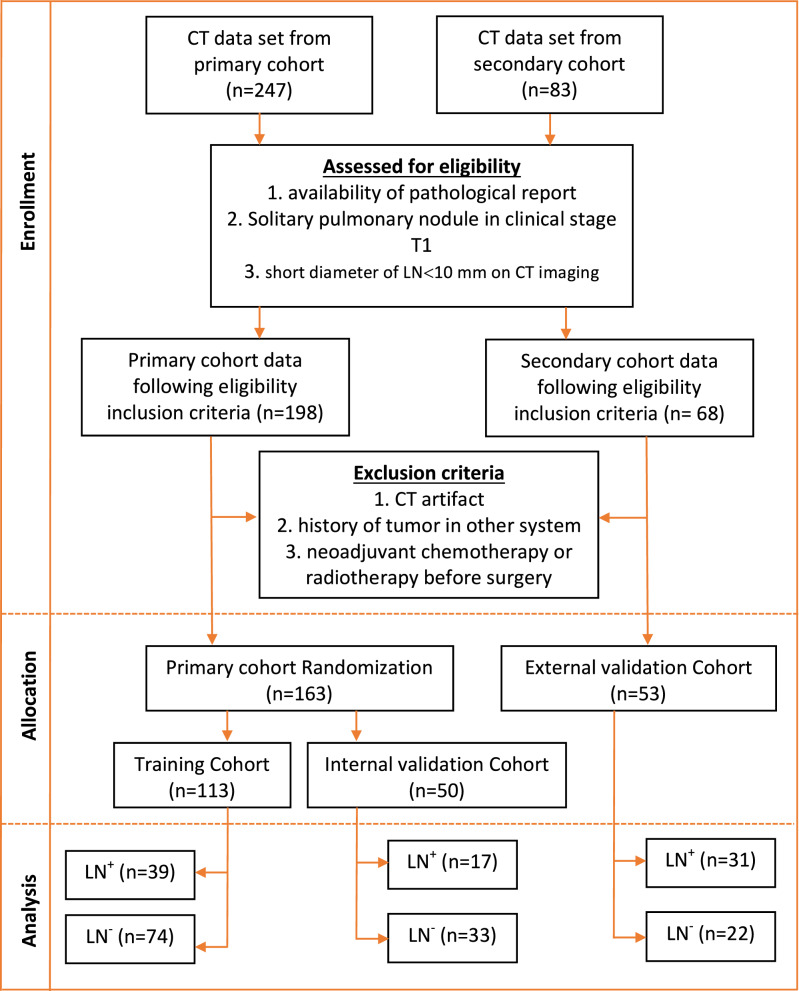
Consolidated standards of reporting trials, or CONSORT, flow diagram of patient enrollment, eligibility, and exclusion criteria of data set.

This study also included a secondary cohort of patients from another hospital (Nanchong Central Hospital) to form an external validation cohort. These patients were enrolled retrospectively from January 2018 to December 2019 using the same criteria as that for the primary cohort. In total of 53 patients [mean age ± standard deviation (SD) 61.37 ± 11.70; range 35–73; male 30; female 23] with 53 tumors were identified and comprise the external validation cohort. The flow diagram of patient enrollment, eligibility, and exclusion criteria is shown in Fig. 1.

### Pre-operative clinical features

The following clinical features were collected for each patient from the medical records: age, gender, smoking status, the maximal diameter of the tumor, and carcinoembryonic antigen (CEA) levels. Laboratory analysis of CEA was done via routine blood tests within one week before surgery. The threshold value of CEA level was 5.0 ng/mL according to the normal range used at our institution.

### Surgical procedures and pathological diagnosis

All patients underwent either lobectomy or sub-lobectomy with systematic lymph node dissection in the same manner. At least six lymph nodes were dissected in accordance with the European Society of Thoracic Surgeons guidelines^30^. The pathological specimens were analyzed by experienced pulmonary pathologists. Pathologic lymph node stage was classified according to the 8th TNM classification in lung cancer^28^.

### Acquisition of images

#### CT scan protocol

All patients underwent a chest CT scan at the respective hospitals before the operation. Contrast enhanced chest CT scan at both the hospitals were performed extending from the lung apex to the adrenal glands at full inspiration. All patients at affiliated Hospital of North Sichuan Medical College underwent scanning with a multi-detector CT (MDCT) scanner (Discovery CT750HD; GE Healthcare, Milwaukee, WI) with the following parameters: tube voltage, 120 kVp; tube current, 250 mAs; scan thickness, 1.25 mm; and interval, 1.25 mm; and pitch, 0.75–1.5. All patients at Nanchong Central Hospital were scanned on MDCT Somatom Definition AS (Siemens Healthineers, Erlangen, Germany) scanner with following acquisition parameter: tube voltage, 120 kVp; tube current, 100–200 mAs; scan thickness, 1.5 mm; and interval, 1.5 mm; and pitch, 0.75–1.5.

### CT semantic features

Two radiologists (B.L. and XF with eight and five years of experience respectively), who were blinded to the clinical pathological findings noted semantic features of each of the lesions. Five CT morphology characteristics for each mass were included: (a) attenuation, (b) spiculation, (c) lobulation, (d) pleural retraction, (e) air bronchogram, and (f) vacuole. Lesion attenuation was divided into three types: pure ground-glass opacity (GGO), part solid, and pure solid. In case of any discrepancy, the final consensus was reached by group discussion.

### Radiomic analysis

#### Image pre-processing

First the linear interpolation of the imaging data to isotropic voxel spacing was carried out to allows for a better comparison of heterogeneous, multi-institutional imaging data. Images were up-sampled to a 1.0 × 1.0 × 1.0 mm^3^ voxel from the original image voxel spacing of 1.0 × 1.0 × 3.0 mm^3^. Then, the Gaussian filter was applied for denoising. To assess the impact of the intensity discretization method on textural features, fixed bin number (FBN) method using 32 bins was implemented. The FBN method discretizes every voxel intensity from a VOI to a fixed number of Ng bins (here 32 bins). It is defined as follows:

$$ X_{d,k} = \left\{ {\begin{array}{*{20}l} {\left| {N_{g} \frac{{X_{gl,k} - X_{gl,min} }}{{X_{gl,max} - X_{gl,min} }}} \right| + 1},&\quad {X_{gl,k} < X_{gl,max} } \\ {Ng}, & \quad {X_{gl,k} = X_{gl,max} ,} \\ \end{array} } \right. $$

where N_g_ corresponds to the fixed number of bins between X_gl,min_ and X_gl,max_, which are the minimum and maximum intensities of the ROI, respectively^31^.

#### Segmentation of lesion of interest

Three dimensional region of interest (3D ROI, i.e. VOI) for GTV as well as lymph node was manually segmented by a single board-certified cardio-thoracic radiologist (B.L. with eight years of experience) across all of the two-dimensional sections of the nodule and LN, with a hand- annotation tool in axial view by using an open-source software ITK‐SNAP (version 3.6.0; http://www.itksnap.org/pmwiki/pmwiki.php?n=Downloads.SNAP3)^32^. The radiologist was blinded to pathologic diagnosis. The radiologist was given the option to vary the window and level setting within this software to efficiently annotate the nodule. For PTV segmentation, GTV was dilated in three dimensions uniformly to capture the region outside the nodule up to a radial distance of 5.0 mm^33^. The intra-nodular mask was then subtracted from this dilated mask to obtain a ring of lung parenchyma immediately around the nodule. This served as a PTV.

#### Extraction of radiomic features

Radiomic features were extracted from each region of interest using Artificial Intelligence Kit software (A.K. software; GE Healthcare, China). The features consisted of six classes of radiomic features: (1) Shape features; (2) First order statistical features; (3) Gray level co-occurrence matrix (GLCM) features; (4) Gray-level size zone matrix (GLSZM) based features; (5) Gray level run length matrix (GLRLM) features; and (6) Inverse difference moment (IDM). The algorithm of each radiomic feature was based on the image biomarker standardization initiative (IBSI).

#### Standardization of the extracted radiomic features

Standardization of the extracted features was applied, using Z-score method, as the images were from two different scanners with different imaging protocols. Both training and validation data were standardized as the min–max normalization, where each feature was normalized as the range from 0 to 1.

$$ Z{ }\,score = \frac{{\left( {x - {\upmu }} \right)}}{{\upsigma }}, $$

where, *x* is the value of feature, μ indicates the average value of this feature for all patients in the cohort, and σ represents the corresponding standard deviation^31^.

#### Feature selection

To assess for segmentation variability, two additional readers (Observer 1: KWF and Observer 2: LX), with six and four years of experience in thoracic imaging respectively were recruited to independently segment a random cohort of 75 nodules and LNs. Observer one conducted lesion segmentation twice, while observer two conducted segmentation once. The inter- and intra-observer reproducibility were assessed using the intra-class correlation coefficients (ICCs). The features with ICC lower than 0.75 were adjudged to have poor agreement and therefore were excluded^34^.

After the ICC selected the repeatable features, spearman correlation analysis (SPM) combined with the least absolute shrinkage and selection operator (LASSO) method were utilized to select the most useful predictive features in the training cohort. The threshold of the Spearman correlation coefficient was 0.9 to reduce feature redundancy^35^, and the LASSO was used to further select the features with penalty parameter tuning that was conducted by tenfold cross-validation based on minimum criteria.

#### Construction of radiomic signature model

Radiomic models were then constructed by multivariable Logistic regression model with the selected radiomic features. Radiomic signatures also called Radiomic Score (Rad-score) were then calculated in training and validation cohort via a linear combination of selected features weighted by their respective coefficients in the models respectively.

$$ {\text{Radiomic signature }}\left( {\text{Rad - score}} \right) \, = \mathop \sum \limits_{i = 1}^{n} C_{i} X_{i} + b, $$

where b is the intercept, $X_{i}$ is the value of ith selected feature and ${C}_{i}$ is the coefficient of the i th selected feature^35^.

In this way, independent radiomic signatures based on GTV, PTV and LN features were obtained. In addition, these radiomic signatures were combined via logistic regression model to build combined radiomic signatures GPTV radiomic signature (GTV + PTV) and GPTV + LN radiomic signature (GTV + PTV + LN).

### Selection of clinical parameters

Clinical features and CT semantic feature are together referred to as clinical parameters hereafter in the present study. Univariate logistic analysis was carried out to select the clinical parameters which were predictive of LN metastasis. Clinical parameters with two-sided p < 0.05 were consider predictors of LN metastasis and were selected.

### Construction and validation of nomogram

Radiomic signatures and clinical parameters were evaluated by univariable logistic regression analysis for prediction of LN metastasis in a training cohort. Variables with < 0.05 were further analyzed by multivariable logistic regression. Finally, an integrative nomogram was built combining the radiomic signatures and clinical parameters identified as independent predictors of LN metastasis in multivariable logistic regression analysis. The discrimination performance of the radiomic signatures and nomogram was evaluated by receiver operating characteristic (ROC) curve analysis and quantified by the area under the ROC curve (AUC). The predictive capability of the radiomic signatures and nomogram were validated on internal as well as external validation cohorts.

### Statistical analysis

R version 3.4.2 (R Foundation for Statistical Computing, Vienna, Austria) (http://www.r-project.org/) was used to carry out all the statistical analyses. The clinical parameters between the groups were compared by using the independent samples Student’s T test (or Wilcoxon Mann–Whitney U test if required) for continuous variables and chi-square test (or Fisher’s exact if required) for categorical variables. Univariate and multivariate logistic regression analyses were performed to determine the predictors of LN metastasis.

Spearman correlation test was applied to remove the high-dimensional feature redundancy. The “glmnet” package of R software was applied to conduct LASSO logistic regression model analysis. The nomogram and calibration curve were constructed by using the “rms” package of R software. Calibration curve was used to analyze the calibration of the radiomics nomogram. The Hosmer–Lemeshow goodness-of-fit test was used to evaluate the model’s fit^36^. The discrimination performance of the radiomics signatures and nomogram in both cohorts were evaluated with ROC curves analysis and quantified by the area under the ROC curve (AUC). The AUCs of the radiomics signatures and the nomogram in the two cohorts were compared by using the DeLong test to evaluate whether over fitting occurred. In addition, to further verify the reliability of the model, bootstrap validation was performed.

By repeating 1000 times bootstrap respectively, the overall accuracy of the model was estimated by Equation:

$$ {\text{Acc}}_{{{\text{overall}}}} = \mathop \sum \limits_{{{\text{i}} = 1}}^{{\text{k}}} \left( {0.632 \times {\text{Acc}}_{{{\text{testset}}}} + 0.368 \times {\text{Acc}}_{{{\text{trainset}}}} } \right). $$

Decision curve was plotted to evaluate the diagnostic efficiency of the model by calculating the net benefits at different threshold probabilities^37^. All statistical tests were two- sided, and P values of < 0.05 were considered statistically significant.

## Results

### Clinical characteristics

In the training cohort, there were 39 patients (34.5%) who had a lymph node metastasis (LN positive) while 74 patients (65.5) without lymph node metastasis (LN negative) on pathological examination. In the internal validation cohort, there were 17 patients (34.0%) with LN positive and 33 (66.0%) with LN negative lymph node status. LN metastases prevalence between the training and internal validation cohorts was insignificant (p = 0.95). Similarly, there were 31 patients (58.5%) with LN positive and 22 (41.5%) with LN negative lymph node status in the external validation group. LN metastases prevalence between the training and external validation group was insignificant (p = 0.63). The baseline clinical features and CT semantic features of the tumor for each cohort according to LN metastasis are given in Table 1.

**Table 1:** Clinical arameters of patients on training, internal and external validation cohort.

Variables	Training cohort (n = 113)		Estimated risk^λ^	p value^‡^	Internal Validation Cohort (n = 50)		Estimated risk^λ^	p value^‡^	External Validation Cohort (n = 53)		Estimated risk^λ^	p value^‡^
	pLN (−) (n = 74)	pLN (+) (n = 39)			pLN (−) (n = 33)	pLN (+) (n = 17)			pLN (−) (n = 22)	pLN (+) (n = 31)		
**Age**												
≤ 60	37 (50.0)	19 (48.7)	1		7 (21.2)	8 (47.1)	1		8 (36.4)	13 (41.9)	1	
> 60	37 (50.0)	20 (51.3)	1.05 (0.48–2.29)	0.90	26 (78.8)	9 (52.9)	0.30 (0.08–1.07)	0.06	14 (63.6)	18 (58.1)	0.71 (0.22–2.28)	0.56
**Gender**												
Male	45 (60.8)	21 (53.8)	1		23 (69.7)	9 (52.94)	1		15 (68.2)	15 (48.4)	1	
Female	29 (39.2)	18 (46.2)	1.33 (0.61–2.91)	0.48	10 (30.3)	8 (47.1)	2.04 (0.61–6.84)	0.25	7 (31.8)	16 (51.6)	2.14 (0.67–6.86	0.20
**Smoking status**												
Never	38 (51.4)	18 (46.2)	1		21 (63.6)	8 (47.1)			13 (59.1)	17 (54.8)	1	
Current/former	36 (48.6)	21 (53.8)	1.23 (0.57–2.68)	0.60	12 (36.4)	9 (52.9)	1.97 (0.60–6.45)	0.26	9 (40.9)	14 (45.2)	1.32 (0.42–4.15)	0.63
**CEA (μg/L)**												
≤ 5	62 (83.8)	23 (58.9)	1		26 (78.8)	13 (76.4)	1		19 (86.4)	20 (64.5)	1	
5–20	12 (16.2)	12 (30.8)	2.70 (1.06–6.85)	0.04*	7 (21.2)	2 (11.8)	0.57 (0.10–3.15)	0.52	3 (13.6)	9 (29.0)	4.00 (0.75–21.35)	0.10
> 20	0 (0.0)	4 (10.3)	/	0.98	0 (0.0)	2 (11.8)	/	0.99	0 (0.0)	2 (6.5)	/	0.99
Tumor size	2.09 ± 0.59	2.42 ± 0.58	2.74 (1.31–5.70)	0.005*	2.17 ± 0.62	2.74 ± 0.31	10.19 (2.01–51.72)	< 0.001*	2.07 ± 0.61	2.51 ± 0.54	3.76 (1.28–11.01)	0.02*
**Tumor location**												
Central	20 (27.0)	16 (41.1)	1		9 (27.3)	9 (52.9)	1		6 (27.3)	14 (45.2)	1	
Peripheral	54 (73.0)	23 (58.9)	0.53 (0.23–1.21)	0.13	24 (72.7)	8 (47.1)	0.33 (0.10–1.13)	0.08	16 (72.7)	17 (54.8)	0.38 (0.11–1.33)	0.13
**Lung lobes**												
RUL	21 (28.4)	9 (23.1)	1		14 (42.4)	3 (17.6)	1		5 (22.7)	8 (25.8)	1	
RML	2 (2.7)	1 (2.6)	1.17 (0.09–14.56)	0.90	3 (9.1)	3 (17.6)	4.67 (0.61–35.49)	0.14	2 (9.1)	3 (9.7)	0.71 (0.07–6.92)	0.77
RLL	21 (28.4)	7 (17.9)	0.78 (0.24–2.48)	0.67	2 (6.1)	2 (11.8)	4.67 (0.46–47.63)	0.19	4 (18.2)	5 (16.1)	0.89 (0.16–5.11)	0.90
LUL	18 (24.3)	13 (33.3)	1.68 (0.58–4.85)	0.33	10 (30.3)	5 (29.4)	2.33 (0.45–12.09)	0.31	8 (36.4)	8 (25.8)	0.71 (0.16–3.23)	0.66
LLL	12 (16.2)	9 (23.1)	1.75 (0.55–5.61)	0.35	4 (12.1)	4 (23.5)	4.67 (0.72–30.12)	0.10	3 (13.6)	7 (22.6)	2.50 (0.36–17.50)	0.36
**Lesion attenuation**												
Solid	54 (72.9)	35 (89.7)	1		23 (69.7)	17 (100.0)	1		16 (72.7)	29 (93.5)	1	
Part solid	7 (9.5)	1 (2.6)	0.22 (0.03–1.87)	0.17	7 (21.2)	0 (0.0)	/	0.99	4 (18.2)	0 (0.0)	/	0.99
GGO	13 (17.6)	3 (7.7)	0.36 (0.09–1.34)	0.13	3 (9.1)	0 (0.0)	/	0.99	2 (9.1)	2 (6.5)	0.29 (0.02–3.40)	0.32
**Lobulation**												
Present	48 (64.9)	30 (76.9)	1		22 (66.7)	11 (64.7)	1		10 (45.5)	22 (71.0)	1	
Absent	26 (35.1)	9 (23.1)	0.55 (0.23–1.34)	0.19	11 (33.3)	6 (35.3)	1.09 (0.32–3.73)	0.89	12 (54.5)	9 (29.0)	0.35 (0.11–1.13)	0.08
**Spiculation**												
Present	37 (50.0)	32 (82.1)	1		22 (66.7)	13 (76.5)	1		13 (59.1)	24 (77.4)	1	
Absent	37 (50.0)	7 (17.9)	0.22 (0.09–0.56)	0.001*	11 (33.3)	4 (23.5)	0.61 (0.16–2.34)	0.48	9 (40.9)	7 (22.6)	0.35 (0.10–1.21)	0.10
**Pleural retraction**												
Present	32 (43.2)	25 (64.1)	1		17 (51.5)	12 (70.6)	1		9 (40.9)	20 (64.5)	1	
Absent	42 (56.8)	14 (35.9)	0.43 (0.19–0.95)	0.04*	16 (48.5)	5 (29.41)	0.44 (0.13–1.54)	0.20	13 (59.1)	11 (35.5)	0.39 (0.12–1.25)	0.11
**Air bronchogram**												
Present	31 (41.9)	26 (66.7)	1		15 (45.4)	9 (52.9)	1		7 (31.8)	14 (45.2)	1	
Absent	43 (58.1)	13 (33.3)	0.36 (0.16–0.81)	0.01*	18 (54.6)	8 (47.1)	0.74 (0.23–2.39)	0.62	15 (68.2)	17 (54.8)	0.49 (0.15–1.63)	0.25
**Vacuole**												
Present	25 (33.8)	13 (33.3)	1		6 (18.2)	4 (23.5)	1		6 (27.3)	12 (38.7)	1	
Absent	49 (66.2)	26 (66.7)	1.02 (0.45–2.32)	0.96	27 (81.8)	13 (76.5)	0.72 (0.17–3.01)	0.65	16 (72.7)	19 (61.3)	0.51 (0.15–1.79)	0.29

*pLN (−)* pathologically lymph node negative, *pLN (+)* pathologically lymph node positive, *CEA* carcinoembryonic antigen, *GGO* ground glass opacity, *RUL* right upper lobe, *RML* right middle lobe, *RLL* right lower lobe, *LUL* left upper lobe, *LLL* left lower lobe.

^λ^Odd ratio with univariate test; ^‡^Chi-square test or Mann–Whitney test; * p < 0.05.

### Feature selection and radiomic signature building

**Figure 2:**
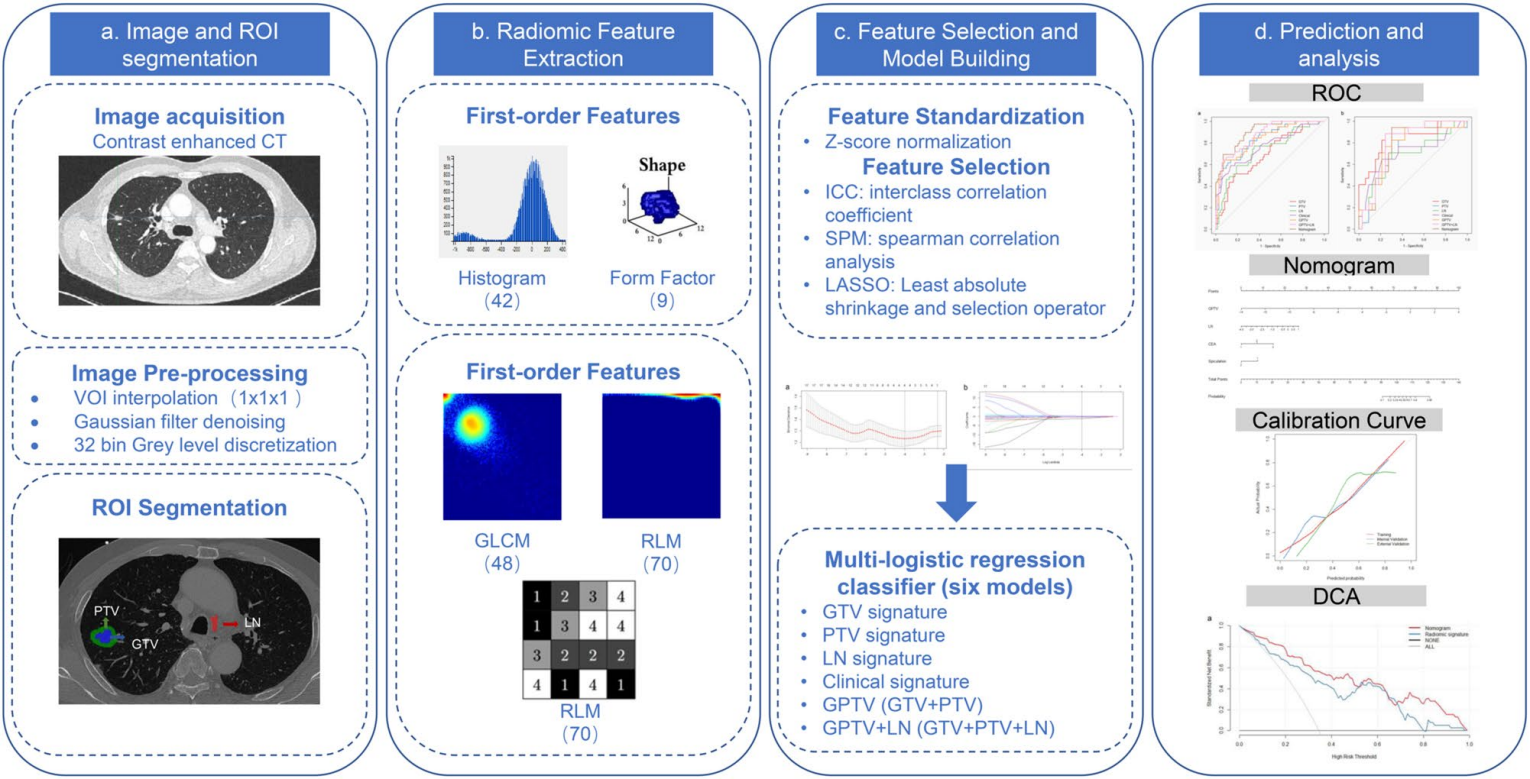
Work flow of tumor segmentation, feature extraction and signature building. Region of interest (ROI) was manually placed on axial CT over gross tumor volume (GTV) (in blue), and lymph nodes (LN) (in red). GTV was dilated 5 mm in all three dimensions uniformly to capture the peritumoral volume (PTV) (green).

Work flow of tumor segmentation, feature extraction and signature building is illustrated in Fig. 2. The total number of features extracted for each signature along with the formulas are shown in Supplementary Table S1. Total of 396 features were extracted from each GTV, PTV, GPTV, and LN. Low reproducible radiomic features, i.e., features with intra- or inter-observer ICC of < 0.75, were considered less reproducible and were excluded. So, the number of GTV, PTV, GPTV, and LN features was reduced to 266, 395, 395, and 155 respectively. Subsequently, redundant features as per Spearman rank correlation coefficients were also excluded. This left 133, 62, 71, and 24 features in GTV, PTV, GPTV, and LN respectively. After that, using LASSO logistic regression model, two, seven, and three features with non-zero coefficients in GTV, PTV, GPTV, and LN respectively were selected. Finally, these features were used by LASSO logistic regression model to build radiomic signatures in a training cohort. GTV, PTV, GPTV, and LN radiomics signature were thus acquired. Furthermore, all the GPTV and LN radiomic features were placed together to create a combined GPTV + LN radiomic signature.

Texture feature selection using the LASSO binary logistic regression model is shown in Supplementary Fig. S1.

### Predictive performance of radiomic signature

The potential association of the radiomics signature with LN status was first assessed in the training cohort and then validated in the internal as well as external validation cohort. In the training cohort, the AUC for GTV, PTV, GPTV, LN, and GPTV + LN was 0.75 (95% CI 0.66–0.85), 0.77 (95% CI 0.76–0.91), 0.84 (95% CI 0.76–0.91), 0.73 (95% CI 0.63–0.83), 0.87 (95% CI 0.80–0.93) respectively. In the internal validation cohort, the AUC for GTV, PTV, GPTV, LN, and GPTV + LN was 0.76 (95% CI 0.60–0.91), 0.72 (95% CI 0.57–0.87), 0.76 (95% CI 0.62–0.91), 0.68 (95% CI 0.52–0.85), 0.78 (95% CI 0.65–0.91) respectively. There was no significant difference in AUCs of radiomic signatures between the two cohorts (DeLong test, p > 0.05 for each comparisons). Upon external validation, the AUC for GTV, PTV, GPTV, LN, and GPTV + LN was 0.74 (95% CI 0.60–0.88), 0.72 (95% CI 0.57–0.87), 0.75 (95% CI 0.61–0.89), 0.64 (95% CI 0.48–0.80), 0.76 (95% CI 0.61–0.91) respectively. Furthermore, there was no significant difference in AUCs of radiomic signatures between the external validation and training cohorts (DeLong test, p > 0.05 for each comparisons). The result of DeLong test is given in Supplementary Table S3 and S4.

**Figure 3:**
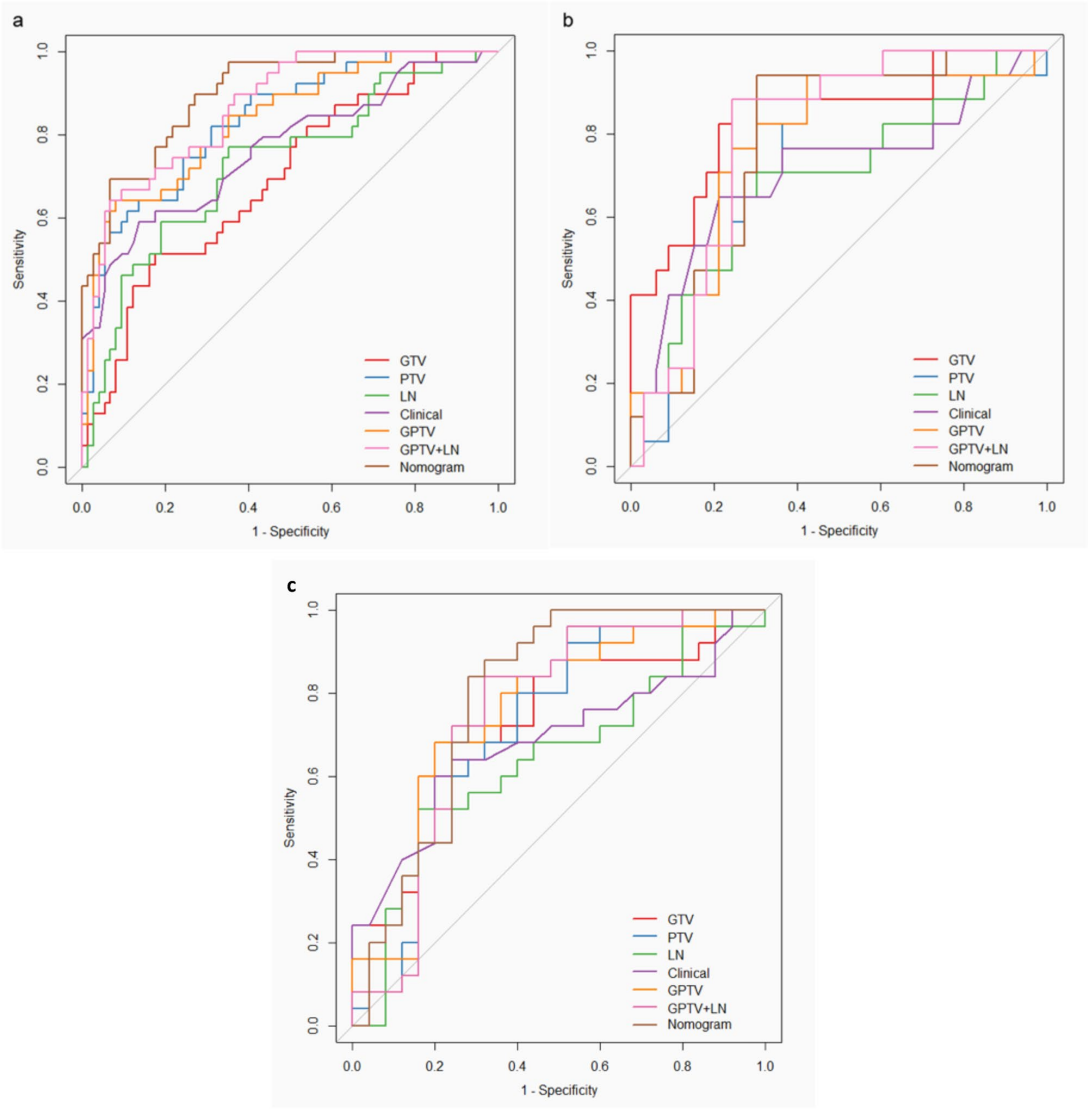
The receiver operating characteristic (ROC) curves of radiomic signatures: (**a**) training cohort, (**b**) internal validation cohort (**c**) external validation.

GPTV + LN radiomic signature had the best AUC in all the cohort (0.87 in the training cohort, 0.78 in the internal and 0.76 in the external validation cohort) in predicting lymph node metastasis. The predictive performance of each individual radiomic signatures on training and validation cohort is listed in Table 2. The ROC curves for each individual signature on the training and validation cohort is shown in Fig. 3.

**Table 2:** Diagnostic performance of radiomics signatures and nomogram.

Signatures	Training cohort			Internal validation cohort			External validation cohort		
	Sensitivity	Specificity	AUC (95% CI)	Sensitivity	Specificity	AUC (95% CI)	Sensitivity	Specificity	AUC (95% CI)
GTV	0.67	0.76	0.75 (0.66–0.85)	0.71	0.73	0.76 (0.61–0.90)	0.68	0.8	0.74 (0.60–0.88)
PTV	0.90	0.58	0.77 (0.76–0.91)	0.76	0.45	0.72 (0.57–0.87)	0.72	0.6	0.72 (0.57–0.87)
GPTV	0.64	0.92	0.84 (0.76–0.91)	0.71	0.76	0.76 (0.62–0.91)	0.68	0.72	0.75 (0.61–0.89)
LN	0.77	0.65	0.73 (0.63–0.83)	0.71	0.58	0.68 (0.52–0.85)	0.68	0.46	0.64 (0.48–0.80)
Clinical	0.59	0.86	0.77 (0.67–0.86)	0.65	0.76	0.71 (0.54–0.88)	0.6	0.8	0.685 (0.53–0.84)
GPTV + LN	0.64	0.93	0.87 (0.80–0.93)	0.71	0.73	0.78 (0.65–0.91)	0.72	0.76	0.76 (0.61–0.91)
Nomogram	0.90	0.73	0.90 (0.84–0.96)	0.94	0.51	0.79 (0.67–0.92)	0.92	0.56	0.79 (0.66–0.93)

*GTV* gross tumor volume, *PTV* peritumoral volume, *GPTV* gross and peritumoral volume, *LN* lymph node, *AUC* area under curve.

### Development of nomogram

**Figure 4:**
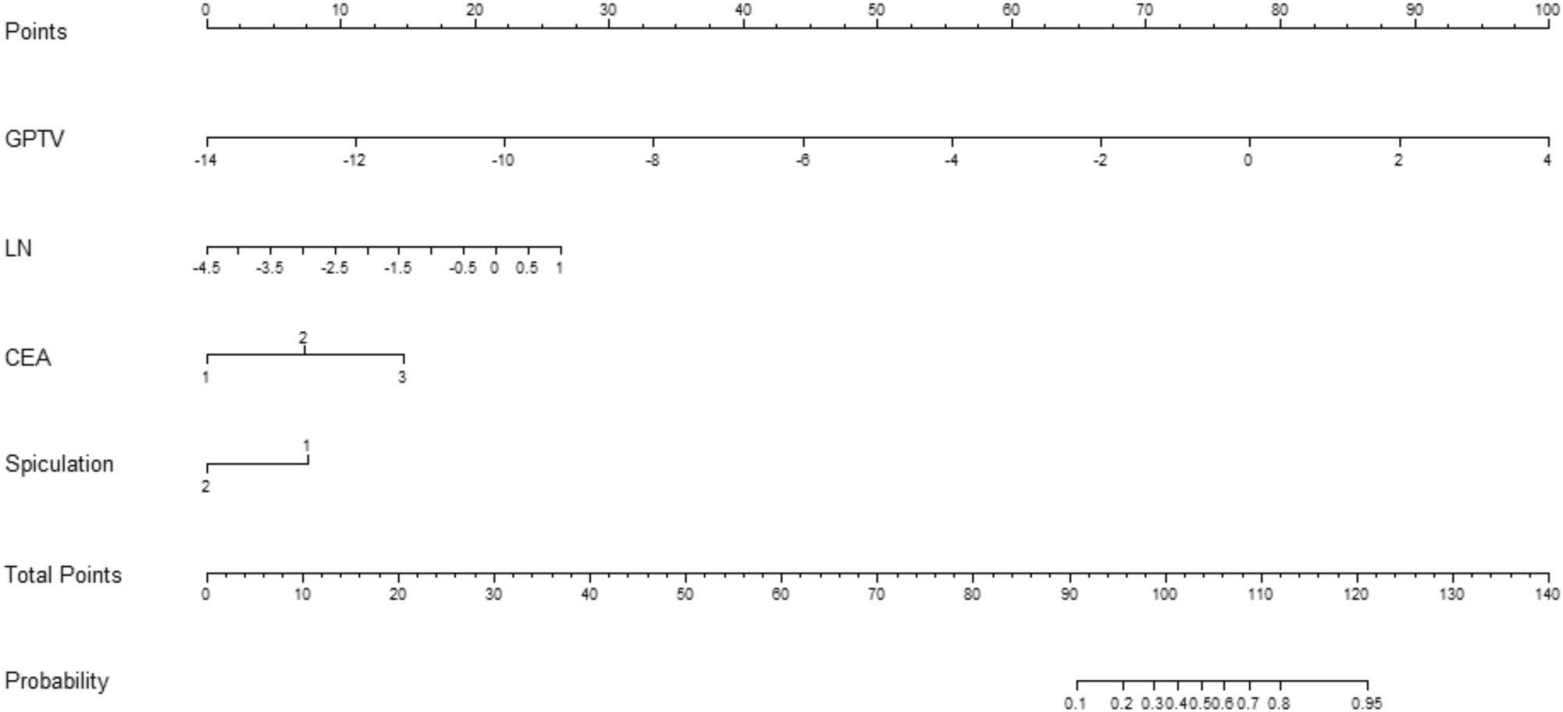
An integrative nomogram incorporating carcinoembryonic antigen (CEA), tumor spiculation and CT radiomics features extracted from gross tumor volume (GTV), peritumoral volume (PTV) and lymph nodes (LN) for the prediction of LN metastasis in patients with cT1N0M0 stage lung adenocarcinoma.

In univariate analysis, five clinical parameters namely, CEA, tumor size, spiculation, pleural retraction, and air bronchogram as well as four radiomic signatures (GTV, PTV, GPTV, LN) significantly correlated with the LN metastasis in the training cohort. Multivariable logistic regression analysis was conducted using these five clinical parameters and the four radiomic signatures. Of these features, two clinical features (spiculation and CEA level) and two radiomic signature (GPTV and LN) significantly correlated with the LN metastasis. The results of the univariate and multivariate regression analysis are summarized in Supplementary Table S2. Finally, a nomogram was created incorporating above identified independent predictors. A nomogram was thus created and is shown in Fig. 4.

### Apparent performance of the radiomics nomogram in the training cohort

The AUC of the nomogram for the prediction of LN status was 0.90 (95% CI 0.84–0.96) in the training cohort (Table 2, Fig. 3a). The calibration curve showed that the predictive value of the nomogram for the LN metastasis was in close approximation with observed value indicating good agreement between them (Supplementary Fig. S2). The Hosmer–Lemeshow test yielded a non-significant statistic (p = 0.6) indicating no departure from perfect fit.

### Validation of the radiomics nomogram

The AUC of the nomogram for the prediction of LN status was 0.79 (95% CI 0.67–0.92) in the internal validation cohort and 0.79 (95% CI 0.66–0.93) in the external validation cohort (Table 2, Fig. 3b,c). There was no significant difference in AUCs between training and internal validation cohorts (DeLong test, p = 0.1) (Supplementary Table S3). Similarly, the difference in AUCs between the training and external validation cohorts was also insignificant (DeLong test, p = 0.1) (Supplementary Table S4). Because the discriminative performance of nomogram dropped by 11% in validation cohort as compared to training cohort, further reliability of nomogram was assessed using bootstrapping validation in training cohort. Even after repeating 1000 times bootstrap, the nomogram in training set achieved the overall accuracy of 0.86 compared with the accuracy of 0.90 derived from the entire original dataset. Similarly, the nomogram in internal validation cohort achieved the overall accuracy of 0.75 compared with the accuracy of 0.79 derived from the entire original dataset (Supplementary Table S5). The finding implied that the performances of the nomogram remained satisfactory even after correction by the optimism.

Moreover, good calibration was observed for the prediction of LN metastasis in both the internal and external validation cohort (Supplementary Fig. S2). The Hosmer–Lemeshow test yielded insignificant statistic for both internal validation (p = 0.3) and external validation (p = 0.2) indicating good agreement between predicted risk of LN metastasis and observed outcome.

### Clinical usefulness of the radiomics signature and nomogram

**Figure 5:**
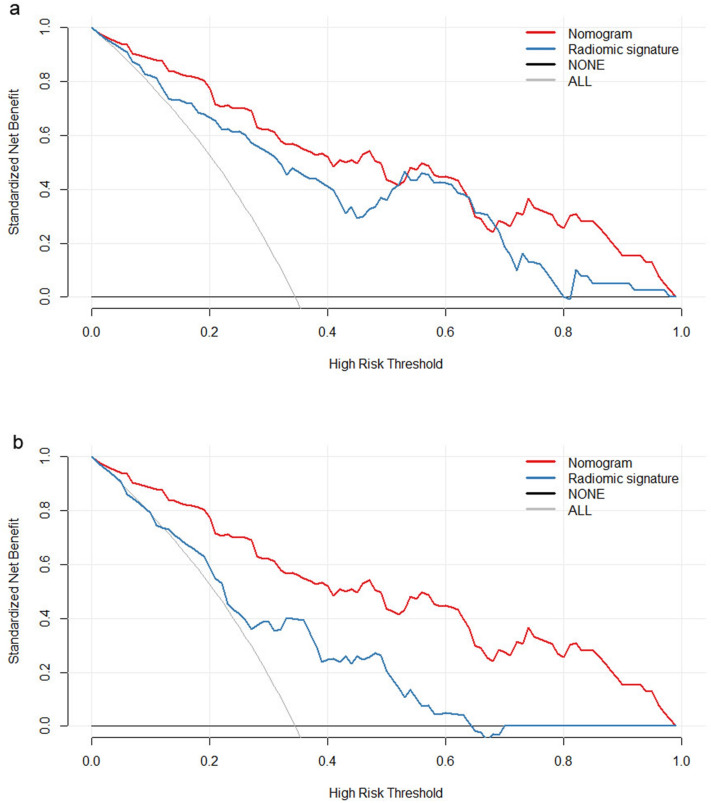
Decision curve analysis (DCA) for the radiomics nomogram along with (**a**) combined gross and peritumoral volume (GPTV) radiomic signature and (**b**) lymph node (LN) radiomic signature. Gray line represents the assumption that all patients have LN metastasis. Black line represents the assumption that all patients have negative LN metastasis. Red curve represents the radiomics nomogram (in both (**a**,**b**). Blue curve represents the GPTV radiomic signature (in **a**) and LN radiomic signature (in **b**). The x-axis shows the threshold probability. The y-axis shows the net benefit. It is clear from the graph that the radiomics signature and nomogram are superior to either treat-all or none strategy within certain ranges of risk.

The decision curve analysis for the radiomics signatures that were identified as independent predictor on the multivariate analysis (GPTV signature and LN signature) and that for the integrative nomogram is shown in Fig. 5. The decision curve demonstrated that the GPTV signatures, LN signature and nomogram would render net gain over the “treat-all-patients” or “treat-none” scheme inside a specific range of threshold (GPTV signature, between five and 80.0%; LN signature between 15 and 65% and nomogram between five and 100%. This suggests that if the threshold probability of a patient or doctor is greater than five percent, the nomogram would add more benefit in predicting LN metastasis than either the treat-all or treat-none scheme.

## Discussion

Herein the present study, a nomogram was developed by incorporating clinical features with radiomics features from GTV, PTV, and LNs. The nomogram, as constructed, had higher AUC than either of the individual radiomic signatures alone suggesting a combination of radiomics features from multiple sources would increase the diagnostic accuracy of model in predicting LN metastasis in cT1N0M0 lung adenocarcinoma.

LN status needs to be accurately determined for selection of optimal surgical treatment, i.e., either systematic LND or SLND in patients with early stage lung cancer^4,5,10^. Although imaging plays a pivotal part in LN staging in clinical setting, it still is challenging to accurately predict LN status by routinely used imaging modalities^38^. Imaging modalities such as CT or FDG-PET/CT. PET/CT, has significantly decreased diagnostic accuracy reported, especially in case where LN size is less than 10.0 mm^39,40^. On the other hand, diagnosis of LN status by CT mainly depends on nodal size criteria which might lead to misdiagnosis, especially in early stage NSCLC in which LNs might be understated without any enlargement on CT imaging^41–43^. Previous studies have found LN size to be unreliable parameter for the evaluation of LN in status NSCLC patients^43,44^.

Emergence of radiomics has given rise to the possibility of precise prediction of LN status by analyzing quantitative features from the primary tumor or LNs. Most of the studies have focused primarily on radiomic features extracted from primary pulmonary tumor to predict the LN status in lung NSCLC^13,20,21,44^. However, recent oncological researches have reported presence of cancerous cells in peritumoral region to have significantly stronger association with distant or local recurrences than their intra tumoral counterparts^45–48^. Studies have demonstrated that cancer cells can microscopically spread beyond grossly visible tumor margin involving peritumoral region^45–48^. Dou et al.^19^ found peritumoral radiomic features to be significantly associated with distant metastasis in NSCLC. In addition, Grove et al.^49^ showed that peritumoral region based radiomic features (i.e., entropy) were higher expressed compared to features extracted from corresponding gross tumour volumes in NSCLC patients. Furthermore, Hosney et al.^50^ developed a deep learning-based prediction model using a 3D convolutional neuronal network for the prediction of OS for NSCLC patients and observed that the network tended to focus on the interface between the tumour and stroma (parenchyma or pleura) regions in the CT images. recent cancer research has shown evidences that extratumoral lung parenchymal tissues surrounding the primary tumor can become involved as cancer infiltrates and metastasizes.

It is believed that hypoxic or necrotic regions preferably appear in the tumor core due to inadequate vascular supply, and the proliferating cancer cells mainly occur in the tumor periphery. Therefore, information from periphery might be crucial stratifying the risk of metastasis. However, only one study, Wang et al.^44^ has considered peritumoral radiomics feature for predicting lymph node metastasis in lung carcinoma and found that incorporation of peritumoral radiomic features with intratumoral features increased the diagnostic performance of the nomogram (GTV radiomic signature verses GPTV radiomic signature: 0.83 verses 0.84). This suggests adding PTV radiomic information could enhance the predictive performance of the radiomic signature.

Furthermore, few studies have used CT texture of LN to discriminate benign ones from malignant LNs in NSCLC with satisfactory results^22,23^. The size of the target lymph nodes in their studies were usually larger than 10.0 mm though. Nonetheless, it does suggest that the information from LNs could well be useful in differentiating benign from malignant LNs. This give rise to an interesting idea of integrating information from both primary tumor and LN to predict LN status. A study from Coroller et al.^51^ demonstrated that adding CT radiomic features extracted from lymph node to the radiomic features from primary pulmonary tumor increased their predictive performance for overall treatment response in NSCLC. However, combined CT radiomics features of both primary tumors and lymph nodes have not been used to predict lymph node metastasis in NSCLC. In the present study, apart from GTV and PTV, we also included features from LN to construct a predictive signature (GPTV + LN) and found it to yielded higher predictive ability when compared to all other individual radiomic signature and found it to yield higher predictive ability when compared to all other individual radiomic signature.

In the present study, among individual signature, GPTV + LN radiomic signature achieved highest AUC in training cohort (GPTV + LN vs GTV vs PTV vs GPTV vs LN: 0.87 vs 0.75 vs 0.77 vs 0.84 vs 0.73). The result was validated on both internal validation (GPTV + LN vs GTV vs PTV vs GPTV vs LN: 0.78 vs 0.76 vs 0.72 vs 0.76 vs 0.68) as well as on external validation cohort (GPTV + LN vs GTV vs PTV vs GPTV vs LN: 0.76 vs 0.74 vs 0.72 vs 0.75 vs 0.64). In addition, in the present study among several clinical parameters, CEA level and speculation (CT sematic feature of tumor) were identified as independent predictor of LN metastasis on multivariable regression analysis. A nomogram was thus built combining these clinical parameters (CEA level and spiculation) with GPTV + LN radiomic signature.

The combined nomogram demonstrated significantly higher AUC when compared to all other individual radiomic signatures in training cohort (Nomogram vs GTV: 0.90 vs 0.75, DeLong test p = 0.03; Nomogram vs PTV: 0.90 vs 0.77, DeLong test p = 0.01; Nomogram vs GPTV:0.90 vs 0.84 DeLong test p = 0.006; Nomogram vs LN: 0.90 vs 0.77, DeLong test p = 0.0006). This implies that combination of GPTV radiomic signature, LN signature and clinical parameters may perform better than a single radiomic signature. On the validation cohort, although not statistically significant, combined nomogram achieved higher AUC compared to all other individual radiomic signatures. This finding suggests that GTV, PTV and LN features could be cooperated to achieve higher predictive performance. However, it is noteworthy that the discriminative performance of nomogram achieved in both the internal (AUC 0.79) and external validation cohort (AUC 0.79) was lower than in the training cohort (AUC 0.90). The observed drop in the performance might be due to random sampling distribution, as the p value calculated by Delong test in training as well as both the validation cohort was greater than 0.05. Therefore, in order to further verify the reliability of the model, bootstrap as an internal validation method was also carried out. By repeating 1000 times bootstrap, the overall accuracy of the nomogram in training set was 0.86 compared with the accuracy (0.90) derived from the entire original dataset. The overall accuracy of the nomogram in internal validation cohort was 0.75 compared with the accuracy (0.79) derived from the entire original dataset. The performances of the nomogram in training and validation cohort yielded a satisfactory optimism indicating that the difference in the performance of the nomogram were not caused by overfitting.

In the present study, the integrative nomogram developed in training cohort achieved higher AUC when compared to all the previous radiomics model studies^13,20–23,44^. This improvement suggests that information integration from multiple sources may reflect that multiple factors of the patient characteristics contributes to a more accurate prediction model.

However, comparison of the discriminative performance of nomogram from the validation cohort needs a careful interpretation as many aforementioned studies either did not have any validation cohort^22,23^ or performed internal validation using resampling method (e.g. bootstrapping or cross-validation)^21^ or split the original dataset non-randomly (e.g., by time or type of CT scanner) to form validation cohort^13,20,44^. Nonetheless, only two studies, Gu et al.^13^, (0.81) and Wang et al.^44^ (0.87) had higher validation AUC compared to the present study (0.79). The difference in discriminative performance of nomogram of internal validation cohort in this study with that of Gu et al.^13^ and Wang et al.^44^ might be due to random sampling approach utilized in this study. The direct comparison of discriminative performance of nomogram of external validation in the present study with that of Gu et al.’s^13^ validation results would be unfair because Gu et al.^13^ had sampled the single center data into validation cohort depending upon time (temporal validation) which is considered as an intermediary between internal and external validation^52^. In contrast, the present study has conducted external validation by collecting data from another hospital where different CT scanner and CT protocol were used. Wang et al.^44^, too had data from single center, spilt into external validation cohort. Only the present study has included data from another center and demonstrated good discriminative capabilities. Moreover, the present study has validated nomogram both internally as well as externally. Furthermore, statistically there was no difference in the discriminative performance of the nomogram between either of validation cohort and training cohort (DeLong Test p > 0.05) suggesting nomogram to have good discrimination in both the validation cohorts as well.

Capability of the nomogram in realizing the necessity of an individual patient to undergo additional treatment, determines the clinical usefulness of the nomogram.

However, the risk-prediction performance, discrimination and calibration, could not capture the clinical consequences of a particular level of discrimination or degree of miscalibration^53,54^. Therefore, to justify the clinical usefulness, whether the radiomics nomogram-assisted decisions would improve patient outcomes or not has to be assessed. Nevertheless, due to disparity in CT image acquisition and clinical data collection, the multi-institutional prospective validation of the nomogram is impractical. Thus, the decision curve analysis was used in the present study. The decision curve, in the present study, showed that if the threshold probability of a patient is more than five percent, the presented nomogram would be more beneficial than either of treat-all-patients or the treat-none scheme to predict LN metastasis.

The present study had several limitations. First, it is a single-institutional and retrospective study which might lead to patient selection bias. Second, the number of patients with cT1N0M0 was limited and in addition, the ratio of LN positive to negative was imbalanced. Third, manual segmentation of the lesion was done which is vulnerable to subjective factors, and fourth, genomic characteristics were not considered. In recent years, to detect LN metastases, increased research with gene markers, such as anaplastic lymphoma kinase (ALK) in patients with clinical N0 lung adenocarcinoma has been proposed^8,55^. In addition, it should be acknowledged that radiomics primarily rely on the extraction, selection, and subsequent classification of predefined features using different machine learning methods alone or in combination. However, there is no “one fits all” approach as performance of various machine learning workflows has been found to depend on application and/or type of data^56^. Machine learning feature extraction and selection is affected by several factors such as imaging scanners, tumor delineation methods, reconstruction methods, discretization, etc.^57,58^. Several methods for image pre-processing, standardization and classification of extracted feature has been proposed to reduce the variability of radiomic features^56^. However, one optimal machine learning approach has not yet been identified. As far as our study is concerned, we have standardized all features using Z-score standardization. Moreover, used an independent validation cohort to assess the prediction performance of nomogram. Future studies not only assessing predictive capabilities but also comparing different feature selection and predictive modeling methods is warranted to decrease dimensionality and reduce overfitting. Moreover, cross-combination of different machine learning method could also be used and compared.

## Conclusion

In conclusion, integrative radiomics nomogram created by combining clinical parameters with radiomic features extracted not only from primary pulmonary tumor but also from peritumoral region and lymph nodes increases the predictive performance of the nomogram in predicting LN status. The present study emphasizes that radiomics feature from both primary tumor as well as the LN should be considered in predicting LN status. An integrative nomogram thus created would offer a feasible and practical reference for individualized management of cT1N0M0 lung adenocarcinoma patients.

## Supplementary Information

Supplementary Information.

## Data Availability

The datasets used and/or analyzed during the current study are available from the corresponding author on reasonable request.

## Abbreviations

LN: Lymph node
GTV: Gross tumor volume
PTV: Peritumoral volume
GPTV: Combined gross and peritumoral volume
ROI: Regions of interest
CT: Computed tomography
HRCT: High-resolution computed tomography
LASSO: Least absolute shrinkage and selection operator
AUC: Area under the curve
NSCLC: Non-small-cell lung cancer
LND: Lymph node dissection
SLND: Selective lymph node dissection
FDG-PET/CT: Fluorine-18 2-fluoro-2-deoxy-d-glucose positron emission tomography/computed tomography
EBUS-TBNA: Endobronchial ultrasound-guided transbronchial needleaspirate
TNM: Tumor-node-metastasis
CEA: Carcinoembryonic antigen
GGO: Ground-glass opacity
GLCM: Gray level co-occurrence matrix
GLSZM: Gray-level size zone matrix
GLRLM: Gray level run length matrix
IBSI: Image biomarker standardization initiative
ICCs: Intra- and inter-class correlation coefficients
ALK: Anaplastic lymphoma kinase
