# Supplementary material for: Integrative nomogram of intratumoral, peritumoral, and lymph node radiomic features for prediction of lymph node metastasis in cT1N0M0 lung adenocarcinomas
Source: Sci Rep. 2021 May 24;11:10829. doi: 10.1038/s41598-021-90367-4 (PMC8144194; doi:10.1038/s41598-021-90367-4)
Supplement: Supplementary file 1 — Supplementary Information. [file 41598_2021_90367_MOESM1_ESM.pdf]

Integrative Nomogram of Intratumoral, Peritumoral, and Lymph Node Radiomic Features for Prediction of Lymph Node Metastasis in cT1N0M0 Lung Adenocarcinomas

1) Sushant Kumar Das#, Ph.D. Interventional Radiology, Department of Interventional Radiology, Affiliated Hospital of North Sichuan Medical College. 63 Wenhua Road, Nanchong, Sichuan, People's Republic of China, 637000. E-mail: sus_mak4u@yahoo.co.in

2) Ke-Wei Fang#, MD Radiology, Department of Interventional Radiology, Affiliated Hospital of North Sichuan Medical College. 63 Wenhua Road, Nanchong, Sichuan, People's Republic of China, 637000. E-mail: 1806973867@qq.com

3) Long Xu, MD Radiology. Department of Interventional Radiology, Affiliated Hospital of North Sichuan Medical College. 63 Wenhua Road, Nanchong, Sichuan, People's Republic of China, 637000. E-mail: 371098997@qq.com

4) Bing Li, MD Radiology. Department of Interventional Radiology, Affiliated Hospital of North Sichuan Medical College. 63 Wenhua Road, Nanchong, Sichuan, People's Republic of China, 637000. E-mail: cbylb@qq.com

5) Xin Zhang, MD, Pharmaceutical Diagnostic team, GE Healthcare, Life Sciences, No. 1 Tongji South Road, Beijing, People's Republic of China, 100176. E-mail: wxyzx666@gmail.com

6) Han-Feng Yang*, Ph.D. Pain management, MD Radiology. Department of Interventional Radiology, Affiliated Hospital of North Sichuan Medical College. 63 Wenhua Road, Nanchong, Sichuan, People's Republic of China, 637000. E-mail: yhfctjr@yahoo.com

# first authors; * corresponding author

Table S1. Radiomics features extracted, selection and formulas of optimal radiomic signatures						
Signatures	Total no. of features	No. of features after selection method			Finally selected Imaging features	Formula
		ICCs	Pearson correlation	LASSO		
GTV	396	266	133	2	SurfaceArea<br>ZonePercentage	GTV=-0.711+0.538*SurfaceArea+0.673*ZonePercentage
PTV	396	395	62	7	HistogramEnergy<br>Correlation_angle135_offset7<br>GLCMEntropy_AllDirection_offset7<br>GLCMEntropy_angle0_offset7<br>InverseDifferenceMoment_angle45_offset7<br>Surface Area<br>Volume CC	PTV=-2.17-3.376*histogramEnergy+0.692*Correlation_angle135_offset7-0.305*GLCMEntropy_AllDirection_offset7-0.286*GLCMEntropy_angle0_offset7-0.689*InverseDifferenceMoment_angle45_offset7+1.857*SurfaceArea-1.448*VolumeCC
GPTV	396	395	71	3	Correlation_angle90_offset7<br>GLCMEntropy_angle135_offset7<br>VolumeCC	GPTV=-1.06+0.807*Correlation_angle90_offset7-1.085*GLCMEntropy_angle135_offset7+0.538
LN	396	155	24	3	Percentile5<br>GLCMEntropy_AllDirection_offset1_SD<br>SurfaceVolumeRatio	LN=-0.748+0.632*Percentile5-0.621*GLCMEntropy_AllDirection_offset1_SD-0.234*SurfaceVolumeRatio

Note: GTV, gross tumor volume; PTV, peritumoral volume; GPTV, gross and peritumoral volume; LN, lymph node; ICC, intraclass and interclass correlation coefficients; LASSO, Least absolute shrinkage and selection operator

Supplementary Files

Table S2: Results of univariate and multivariate regression analysis		
Variables	Univariate	Multivariate
GTV	0.001*	0.514
PTV	0.000*	0.100
GPTV	0.000*	0.000*
LN	0.001*	0.021*
Age	0.897	-
Gender	0.476	-
Smoking status	0.600	-
CEA	0.002*	0.023*
Tumor size	0.007*	0.924
Tumor location	0.131	-
Lung lobes	0.245	
Lesion attenuation	0.070	-
Lobulation	0.191	-
Spiculation	0.001*	0.034*
Pleural retraction	0.037*	0.566
Air bronchogram	0.014*	0.476
Vacuole	0.962	-

Note: GTV, gross tumor volume; PTV, peritumoral volume; GPTV, gross and peritumoral volume; LN, lymph node; CEA, carcinoembryonic antigen; * P < 0.05

Table S3: DeLong Test							
Model	GTV	PTV	GPTV	LN	Clinical	GPTV+LN	Nomogram
GTV	1	0.5	0.9	0.5	0.7	0.8	0.7
PTV	0.6	0.7	0.7	0.7	0.9	0.6	0.5
GPTV	0.2	0.3	0.4	0.5	0.6	0.7	0.5
LN	0.7	0.5	0.1	0.7	0.8	0.3	0.3
Clinical	0.8	1	0.2	0.6	0.6	0.5	0.4
GPTV+LN	0.05	0.09	0.1	0.003*	0.06	0.2	0.7
Nomogram	0.006*	0.01*	0.03*	0.0006*	0.001*	0.1	0.1
Training				Internal Validation			

Note: GTV, gross tumor volume; PTV, peritumoral volume; GPTV, gross and peritumoral volume; LN, lymph node; * P < 0.05

Table S4: DeLong Test							
Model	GTV	PTV	GPTV	LN	Clinical	GPTV+LN	Nomogram
GTV	0.6	0.8	0.9	0.3	0.6	0.9	0.5
PTV	0.6	0.2	0.09	0.5	0.7	0.5	0.2
GPTV	0.2	0.3	0.3	0.3	0.5	1	0.4
LN	0.7	0.5	0.1	0.4	0.7	0.2	0.04
Clinical	0.8	1	0.2	0.6	0.4	0.5	0.2
GPTV+LN	0.05	0.09	0.1	0.003*	0.06	0.1	0.2
Nomogram	0.006*	0.01*	0.03*	0.0006*	0.001*	0.1	0.1
Training				External Validation			

Note: GTV, gross tumor volume; PTV, peritumoral volume; GPTV, gross and peritumoral volume; LN, lymph node; * $P < 0.05$

Table S5: Bootstrap validation			
Cohort	Sensitivity	Specificity	Accuracy
Training	0.85	0.87	0.86
Internal Validation	0.77	0.73	0.75

Note: Bootstrap was repeated 1000 times to derived overall accuracy from original dataset

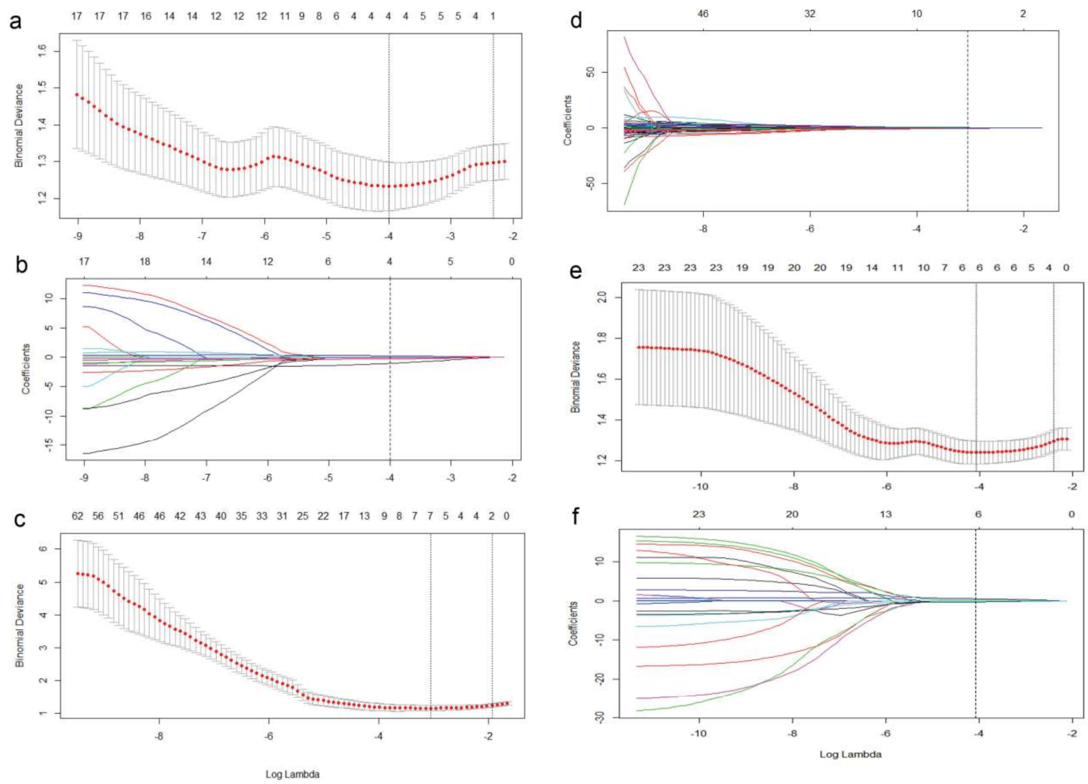

Fig S1: Texture feature selection using the least absolute shrinkage and selection operator (LASSO) binary logistic regression model. (A) Tuning parameter (l) selection in the LASSO model used 10-fold cross-validation via minimum criteria for GTV (a), PTV (c) and LN (e). LASSO coefficient profiles of the 396 texture features of GTV (b), PTV (d) and LN (f)

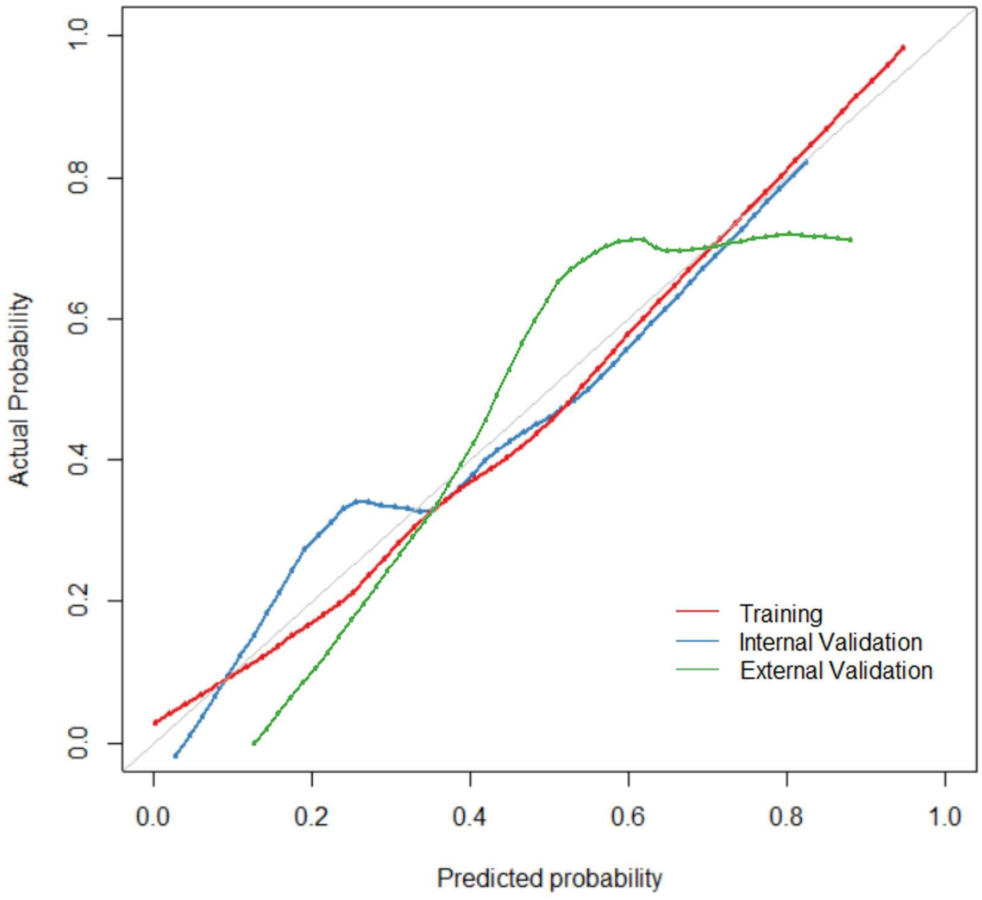

Fig S2: Calibration curves of the nomogram in the training cohort and validation cohorts. The 45° gray line represents a perfect prediction. The predictive performance of the nomogram is represented by blue line in training cohort, red line in internal validation cohort and green line in external validation cohort. All the lines have a close fit to gray line, which indicates good predictive capability of the nomogram in each cohort.
